# Effects of Neuromuscular Electrical Stimulation on Quadriceps Muscle Strength in the Early Postoperative Period after Total Knee Arthroplasty

**DOI:** 10.1298/ptr.E10327

**Published:** 2025-03-10

**Authors:** Shigeki SAKAI, Masanori WATANABE, Yuta ITOH, Nahoko SATO, Naoya HAMAGUCHI, Makoto FUKUTA

**Affiliations:** 1 Department of Rehabilitation, Tajimi Smart Clinic, Japan; 2 Faculty of Rehabilitation Science, Nagoya Gakuin University, Japan

**Keywords:** Neuromuscular electrical stimulation, Quadriceps muscle strength, Total knee arthroplasty, Early postoperative intervention

## Abstract

Objectives: This study aimed to determine whether neuromuscular electrical stimulation (NMES) of the quadriceps muscle early after total knee arthroplasty (TKA) is effective in improving muscle strength. Methods: This was a single-center, non-blinded, randomized controlled trial involving 37 patients (60 knees) who underwent TKA. Patients were randomly assigned to either the intervention group (19 patients, 30 knees) or the control group (18 patients, 30 knees). Both groups received standard rehabilitation starting on postoperative day 1. Additionally, the intervention group received NMES therapy starting on postoperative day 2. NMES was administered at the highest tolerable intensity for 15 min per session (10-s stimulation and 10-s rest) twice daily for 4 weeks. Outcome measures included voluntary isometric quadriceps strength, knee joint range of motion (ROM), visual analog scale (VAS), and the Timed Up and Go (TUG) test, which were assessed preoperatively and at 4, 8, and 12 weeks postoperatively. The outcomes were compared between the two groups. Results: Both groups showed improvements in all outcomes over time. However, no significant differences were observed between the two groups (muscle strength: p = 0.412, flexion ROM: p = 0.668, extension ROM: p = 1.000, VAS score: p = 0.192, TUG test score: p = 0.296) (p-values are main effects of group factors in the split-plot analysis of variance). Conclusions: NMES in the early postoperative period after TKA did not provide significant additional functional recovery benefits compared with standard rehabilitation.

## Introduction

Total knee arthroplasty (TKA) effectively relieves pain and improves function in patients with advanced knee osteoarthritis. However, quadriceps muscle weakness is commonly observed postoperatively^1,2)^. Quadriceps muscle strength has been reported to decrease to 50%–60% of preoperative levels at 1 month after TKA^1,2)^. Other studies have shown that the quadriceps-to-hamstring strength ratio does not return to normal levels as in healthy individuals even 6–13 years after TKA^3)^. Moreover, lower limb muscle weakness is associated with an increased risk of falls^4)^ and a decline in quality of life (QoL)^5)^. Therefore, early recovery and prevention of postoperative muscle strength loss are crucial.

Standardized physical therapy and pharmacological analgesics improve muscle strength and reduce pain after TKA^6)^. However, rehabilitation protocols vary globally^7,8)^. Neuromuscular electrical stimulation (NMES) is considered an effective modality for accelerating recovery after surgery^9)^, and its use dates back to the 18th century^10)^. A systematic review involving 933 participants revealed that NMES is an effective intervention for muscle weakness and is a valuable component of rehabilitation programs^11)^. Some reports have shown that introducing NMES as an adjunct to standard rehabilitation at 1–2 weeks postoperatively is effective in restoring diminished quadriceps strength^12,13)^. However, few studies have been conducted on the introduction of NMES in the early postoperative period to prevent muscle weakness, and no consensus has been reached on its efficacy^14,15)^. Because NMES induces passive muscle contractions, it may be advantageous in the early postoperative period when voluntary movement is limited by pain or weight-bearing restrictions.

Therefore, this study aimed to evaluate the effectiveness of NMES in preventing quadriceps muscle weakness by comparing quadriceps strength at 4, 8, and 12 weeks postoperatively with preoperative strength. For this purpose, NMES was applied to the quadriceps muscle in addition to standard rehabilitation in the early postoperative period following TKA.

## Methods

### Study design and randomization

This study was a non-blinded, randomized, controlled trial designed to evaluate the benefits of applying NMES in addition to the standard rehabilitation program in the early postoperative period. Eligible patients were randomly assigned to the NMES or control group before the preoperative assessment. Randomization was stratified by unilateral or bilateral TKA and sex. Based on an effect size of 0.81 calculated from a previous study^12)^, a sample size of 25 patients per group was required. Recruitment continued until the target number of participants was reached.

### Patients

Patients scheduled for primary unilateral or bilateral simultaneous TKA at the Tajimi Smart Clinic between October 2022 and February 2023 were consecutively recruited. All patients underwent cementless TKA using the medial parapatellar approach performed by a single orthopedic surgeon. The exclusion criteria were inability to provide informed consent, age <50 or >85 years, uncontrolled hypertension, uncontrolled diabetes, body mass index >35 kg/m2, significant neurological disorders, osteoarthritis in the non-operative knee (defined as moderate or greater pain during activity), and any other unstable orthopedic condition in the lower extremities.

This study was conducted in accordance with the principles of the Declaration of Helsinki and was approved by the Medical Research Ethics Committee of Nagoya Gakuin University (Approval No. 2022-01). Patients were informed about the study’s objectives and procedures, the presence or absence of invasive disclosures, and the handling of personal information before enrollment. Written informed consent was obtained from all participants.

### Protocol

**Fig. 1.**
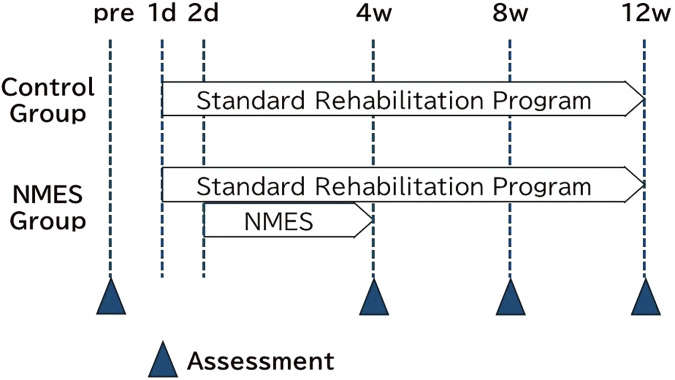
Study protocols

Both groups received a standard rehabilitation program starting on postoperative day 1. Additionally, the NMES group participated in an electrical stimulation program starting on postoperative day 2 for 4 weeks. Both groups were assessed at baseline (preoperatively) and 1, 2, and 3 months postoperatively (Fig. 1).

### Standard rehabilitation

Range of motion (ROM) exercises to restore knee joint mobility and partial weight-bearing exercises for the lower limbs were initiated on the first postoperative day. These exercises can maintain knee flexibility and prevent muscle strength decline from an early stage. Based on the patient’s pain level, strengthening exercises and gait training were gradually introduced on postoperative days 2 to 3. Additionally, stair-climbing exercises were added on days 5–7 to help patients regain independence in ADL (Appendix 1). Patients who underwent unilateral TKA were discharged 8–10 days postoperatively, whereas those who underwent bilateral TKA were discharged between postoperative days 12 and 14. After discharge, the patients were instructed to continue the prescribed exercise program daily (Appendix 1). Additionally, they received weekly outpatient physical therapy sessions to ensure that the exercises were performed correctly, with load adjustments or modifications made as needed.

### NMES

**Fig. 2.**
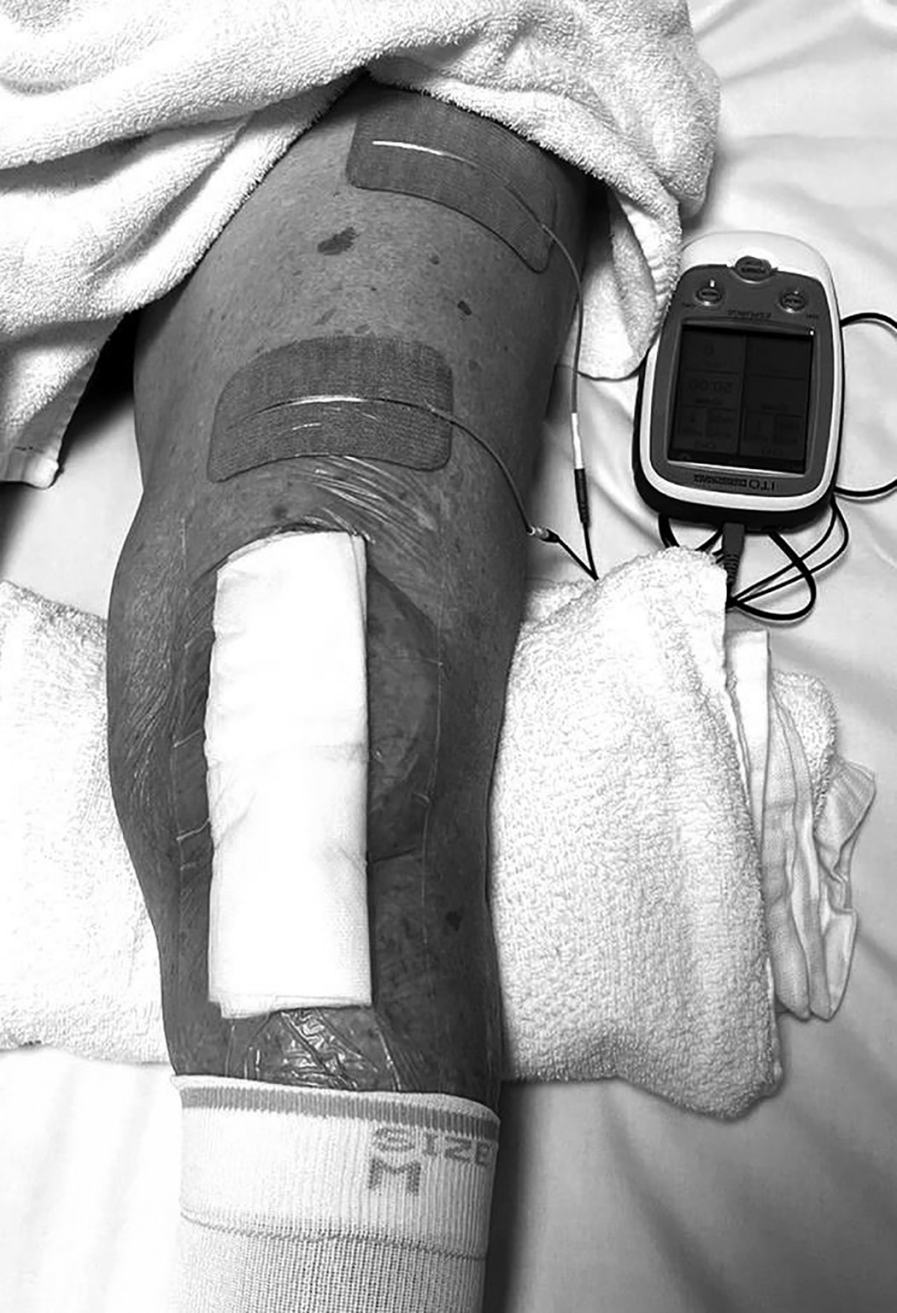
Stimulation location The electrodes were placed on the proximal lateral and distal medial thighs. Patients were placed in a long sitting position with the hip joint flexed to approximately 80° and the knee joint flexed to approximately 30°. They were instructed to voluntarily contract the knee extensor muscles to crush the cushion under the knee during the electrical stimulation.

NMES was performed using an electrical stimulation device (Espurge; Ito, Saitama, Japan) for 15 min per session, with two sessions conducted daily throughout the intervention period. The electrode size is important because it directly influences current density; smaller electrodes can result in a higher current density, leading to painful stimulation before achieving sufficient muscle contraction for strength enhancement. Therefore, large rectangular electrodes (PALS, 5 × 9 cm; Axelgaard Manufacturing, Fallbrook, CA, USA) were used in this study to maximize treatment tolerance. The electrodes were placed on the proximal lateral and distal medial parts of the anterior thigh, and markers were used to ensure consistent reapplication by the participants (Fig. 2). Participants sat with their hips and knees flexed to approximately 80° and 30°, respectively. Participants were instructed to voluntarily contract their knee extensor muscles by pressing on a cushion or pillow placed under the knee during the electrical stimulation.

Electrical stimulation was delivered at a frequency of 40 Hz with a pulse width of 250 ms, with a 10-s on-time (including a 3-s ramp-up period) followed by a 10-s off-time. The intensity was set at a maximum tolerable level during each session, and participants were encouraged to increase the intensity as much as they could tolerate.

The participants learned how to operate the stimulation device during their hospital stay and were able to use it safely and appropriately. After discharge, the condition of the electrodes and participant adherence to the protocol were checked during outpatient rehabilitation visits, and replacement or reinstruction was provided if necessary. NMES training was incorporated into the muscle-strengthening exercises included in the standard rehabilitation program. Therefore, no difference was observed between the two groups in rehabilitation intervention time.

### Assessment

**Fig. 3.**
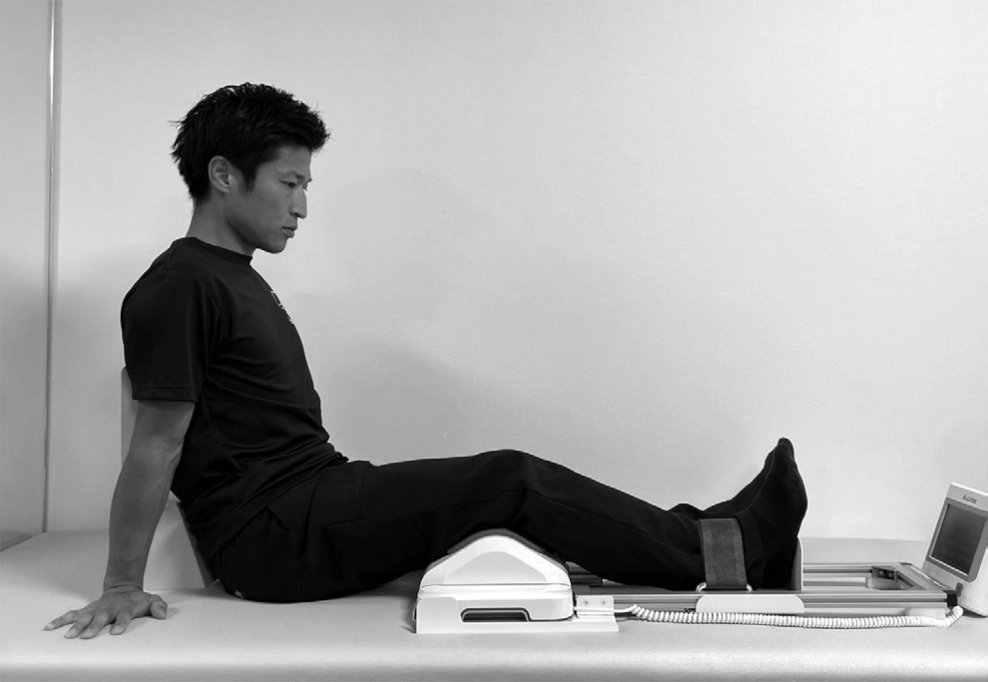
Measurement of knee extensor strength Knee extension was performed by lifting the ankle, which was secured with a belt, while measurements were taken using a sensor placed under the knee.

The primary outcome was the measurement of quadriceps muscle strength. Muscle strength was measured using Locomo Scan-II (ALCARE, Tokyo, Japan) (Fig. 3). This device allows for convenient measurement of isometric quadriceps muscle strength at approximately 30° of knee flexion, and its validity and reliability have been reported during its development stage^16)^. Measurements were performed three times, and the maximum value was used as the representative value. During the assessment, standardized verbal feedback was provided by the evaluator to encourage all participants to exert their maximum quadriceps strength. The secondary outcomes were the visual analog scale (VAS), ROM, and Timed Up and Go (TUG) test. Active knee joint ROM was measured in the supine position using a goniometer. For active knee flexion, participants were instructed to flex their knees as much as possible while maintaining their heels on the supporting surface. Any extension limitation of the knee was reported as a negative value.

### Statistical analysis

Between-group comparisons of preoperative quadriceps muscle strength were performed using values normalized to each participant’s body weight, whereas comparisons of postoperative quadriceps muscle strength were performed using percentage changes from preoperative values (postoperative/preoperative × 100). Temporal comparisons were performed at three postoperative time points: 4, 8, and 12 weeks. Other measurement results, including preoperative data, were temporally compared across all four time points.

Baseline continuous variables were compared between the groups after confirming the normality of the data using the *t*-test or Mann–Whitney U test. The gender ratios of the participants and the targeted limbs were analyzed using the χ-square test. Postoperative comparisons between the groups and the analysis of temporal changes were performed using the split-plot analysis of variance. Statistical analyses were performed using R 4.0.2 for Windows (CRAN, free software). The significance level was set at 5% for all analyses.

## Results

**Fig. 4.**
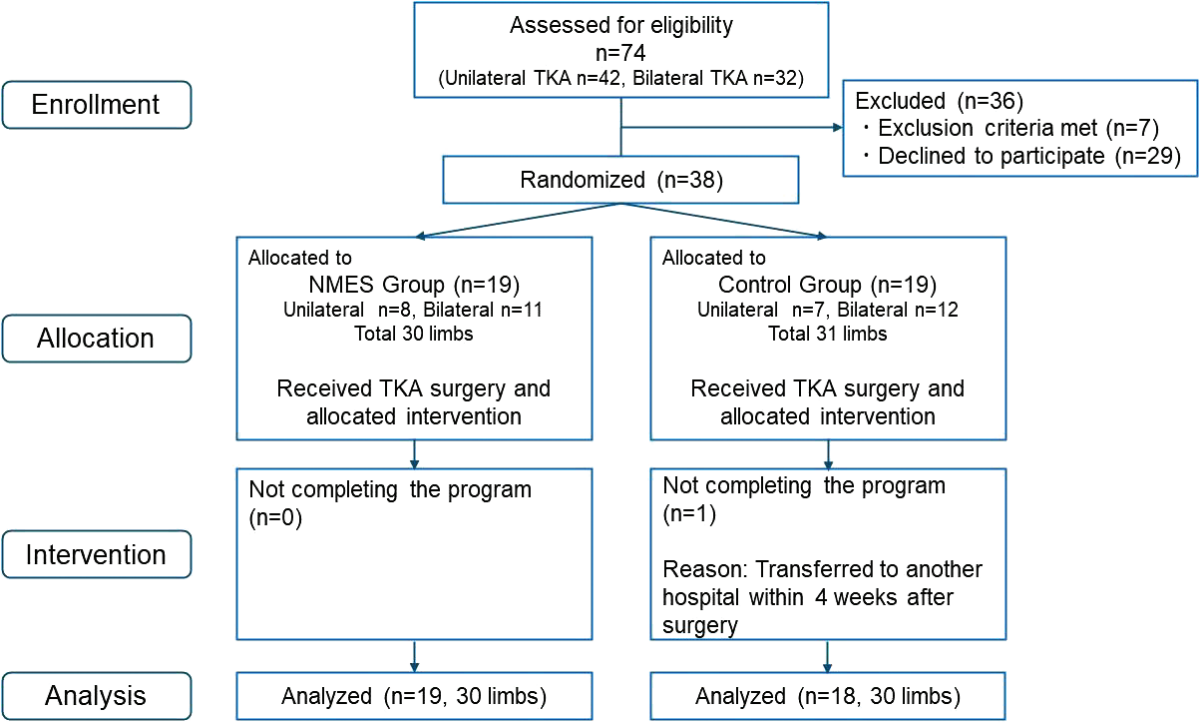
CONSORT flowchart showing the flow of patients through the trial

A total of 74 patients were enrolled in this trial. Among them, 29 refused to participate, and 7 were excluded based on the eligibility criteria. The remaining 38 patients were assigned to the two groups, among whom 37 completed all aspects of the program. Finally, 19 participants (30 knees) in the NMES group and 18 participants (30 knees) in the control group were included in the analysis (Fig. 4).

No significant differences in age or gender ratio were observed between the two groups. Comparison of preoperative measurements showed a significant difference only in VAS scores, which were higher in the control group than in the NMES group (70.6 ± 20.2 vs. 52.2 ± 20.5, p = 0.009) (Table 1).

**Table 1.** Preoperative comparison

	NMES group	Control group	p values
Age	71.4 (7.3)	72.5 (7.3)	0.672
Sex (knees)	M: 3; F: 16	M: 4; F: 14	0.693
	(M: 4; F: 26)	(M: 7; F: 23)	(0.505)
Quadriceps strength (kgf/kg)	0.52 (0.16)	0.46 (0.14)	0.144
Knee flexion (°)	129 (11.9)	123 (11.6)	0.084
Knee extension (°)	−6 (5.0)	−7 (6.2)	0.421
VAS score (mm)	52.2 (20.5)	70.6 (20.2)	0.010*
TUG test score (s)	8.2 (2.1)	9.4 (2.5)	0.097

Values were presented as means (standard deviation).

Continuous variables were analyzed using the *t*-test or Mann–Whitney U test, and nominal variables were analyzed using the χ-square test.

*p < 0.05, Student’s *t*-test.

NMES, neuromuscular electrical stimulation; VAS, visual analog scale; TUG, Timed Up and Go

The percentage changes in muscle strength from baseline were 82.8% at 4 weeks, 95.0% at 8 weeks, and 112.6% at 12 weeks in the NMES group and 89.9% at 4 weeks, 102.8% at 8 weeks, and 114.2% at 12 weeks in the control group. Split-plot analysis of variance indicated a significant increase in muscle strength over time, with differences observed at 4, 8, and 12 weeks (4 vs. 8 weeks, p = 0.000; 4 vs. 12 weeks, p = 0.000; and 8 vs. 12 weeks, p = 0.000). However, no significant differences were observed between the two groups (p = 0.412), and no interaction effect was observed (p = 0.443) (Table 2 and Appendix 2).

**Table 2.** Postoperative changes in each value

	Before		After 4 weeks		After 8 weeks		After 12 weeks	
	NMES group	Control group	NMES group	Control group	NMES group	Control group	NMES group	Control group
Quadriceps strength (%) (post/pre × 100)			82.8 (24.0)	89.9 (29.0)	95 (24.9)^*b*^	102. 8 (35.4)^*b*^	112.6 (28.1)^*b,c*^	114.2 (33.9)^*b,c*^
Knee flexion (°)	128 (11.9)	123 (11.6)	109 (16.0)^*a*^	112 (9.1)^*a*^	114 (13.7)^*a,b*^	117 (9.1)^*a,b*^	117 (13.1)^*b,c*^	121 (10.1)^*a,b,c*^
Knee extension (°)	−6 (5.0)	−7 (6.2)	−5 (4.3)	−5 (4.3)	−5 (3.6)^*a,b*^	−4 (3.6)^*a,b*^	−3 (3.6)^*a,b,c*^	−3 (3.4)^*a,b,c*^
VAS score (mm)	52.2 (20.5)	70.6 (20.2)	37.7 (24.9)	39.2 (20.0)	26.4 (22.2)^*a,b*^	30.2 (23.7)^*a,b*^	19.2 (16.0)^*a,b,c*^	21.8 (19.0)^*a,b,c*^
TUG test score (s)	8.2 (2.1)	9.4 (2.5)	11.9 (5.0)^*a*^	11.8 (3.8)^*a*^	8.7 (2.0)^*b*^	9.4 (2.3)^*b*^	7.8 (1.5)^*b,c*^	9.1 (2.7)^*b,c*^

Values were presented as means (standard deviation).

^*a,b,c*^p < 0.05, analysis of variance for the split-plot factorial design.

^*a*^Significant difference before surgery.

^*b*^Significant difference 4 weeks postoperatively.

^*c*^Significant difference 8 weeks postoperatively.

NMES, neuromuscular electrical stimulation; VAS, visual analog scale; TUG, Timed Up and Go

A significant interaction was observed for knee flexion ROM (p = 0.004). However, post hoc testing revealed no significant differences between the two groups at any time point. Comparisons between time points (repeated measures) showed that both groups exhibited significant differences from preoperative to 8 weeks postoperatively. In comparisons between preoperative and 12 weeks postoperatively, only the control group did not show significant differences (Table 2, Appendix 2).

Additionally, no significant interaction or differences were observed between the two groups in terms of knee extension ROM, VAS score, or TUG test score. Significant differences were observed in repeated measures. A significant improvement in knee extension ROM and VAS score was observed over time compared with preoperative values, whereas the TUG test score showed temporary deterioration at 4 weeks postoperatively but improved to baseline levels from 8 weeks onward (Table 2 and Appendix 2).

## Discussion

In this study, the effectiveness of NMES was not demonstrated. These findings differ from those of a previous study^14)^, which showed that early postoperative NMES was effective in mitigating muscle strength decline. Various studies have investigated the NMES settings, and Nussbaum et al. recommended 10–30 muscle contractions per day^17)^. A previous study implemented 30 muscle contractions per day for 6 weeks and reported the effectiveness of NMES in improving muscle weakness^14)^. By contrast, this study applied 90 muscle contractions per day for 4 weeks. Although the intervention period was short, the applied load was sufficient. Regarding the NMES stimulation cycle (on–off time), a ratio of 1:2 or 1:3 is recommended^17)^. In this study, a 1:1 condition, including a 3-s ramp-up period, was employed, which may have resulted in excessive loading, potentially impeding improvements in muscle strength.

Although previous studies have reported the effectiveness of NMES in promoting muscle strength recovery after targeting only unilateral TKA^12,14)^, our study included unilateral and bilateral TKA, with a higher proportion of patients undergoing bilateral procedures. Regarding weight-bearing on the operated limb after unilateral TKA, the operated limb has been reported to bear approximately 80% of the load compared with the non-operated limb during the sit-to-stand test at 6 weeks postoperatively^18)^, with asymmetry persisting even after a longer duration^19)^. Additionally, the ground reaction force of the operated limb during gait at 3 months postoperatively has been reported to be lower than that of an age-matched control group^20)^. Based on these findings, bilateral TKA was considered less prone to asymmetry in postoperative lower limb loading; although the unilateral TKA was prone to muscle inactivity due to asymmetry in postoperative lower limb loading, and muscle weakness prevention using NMES was significant. Moreover, the resulting voluntary muscle contractions may have made the effect of NMES less noticeable. However, this hypothesis would be refuted if the activity level in bilateral TKA was significantly lower than that in unilateral TKA. Currently, no study has directly compared postoperative activity levels between unilateral and simultaneous bilateral TKA. Therefore, future studies should address this comparison. Improvements in early postoperative mobility (from walker to cane) have been reported to occur a few days earlier in unilateral TKA than in bilateral TKA. Consequently, the discharge date is a few days earlier in unilateral TKA^21)^. However, no differences in the total score or functional activities of the New Knee Society Score^22)^ have been observed at 3 months postoperatively^23)^. In this study as well, while slight differences in activity levels may have been observed during the early postoperative period, it is considered that no significant differences were present throughout the entire study period.

Additionally, differences in positioning during muscle strength measurements should be considered. A previous study that measured the isometric strength of the quadriceps at knee flexion angles of 30°, 60°, and 90° from before surgery to 12 months after surgery reported that strength at 30° did not show a significant difference; however, strength at 60° and 90° significantly decreased at 3 weeks postoperatively compared with preoperative levels^24)^. Furthermore, recovery at deeper flexion angles was slower until 12 months after surgery. All studies that reported significant improvements in muscle strength with NMES intervention measured strength at 60°^14)^ or 90°^12)^ of knee flexion. In this study, muscle strength was measured at an angle where the difference in strength decline was less pronounced, which may explain why the effect of NMES was not observed.

This study has some limitations. This was a non-blinded study. Blinding was not feasible due to practical challenges. We attempted to minimize bias in strength measurements by selecting methods in which direct contact with the measurer was avoided, such as using a handheld dynamometer. Further studies using the prospective randomized open-blinded end-point method are preferable. Additionally, this study evaluated the short-term effects up to 12 weeks postoperatively but did not assess the long-term effects or durability of the intervention. Therefore, further studies with longer follow-up periods are needed to evaluate the effects of NMES. Ultimately, an optimal NMES protocol tailored to each patient can be established to enhance clinical application.

## Conclusions

NMES in the early postoperative period after TKA did not provide significant additional functional recovery benefits compared with standard rehabilitation.

## Acknowledgments

We would like to express our deepest gratitude to the staff of the Tajimi Smart Clinic for their great cooperation.

## Funding

This study was supported by a Grant-in-Aid from Nagoya Gakuin University.

## Conflicts of Interest

There is no conflict of interest to disclose.

## Supplementary Materials

Appendix 1. Standard Rehabilitation Program/Home Exercise Program.

Appendix 2. Detailed results of analysis of variance for split-plot factorial design.
