# Supplementary material for: Effects of Neuromuscular Electrical Stimulation on Quadriceps Muscle Strength in the Early Postoperative Period after Total Knee Arthroplasty
Source: Phys Ther Res. 2025 Mar 10;28(1):54–60. doi: 10.1298/ptr.E10327 (PMC12047048; doi:10.1298/ptr.E10327)
Supplement: Appendix 1. — Standard Rehabilitation Program/Home Exercise Program. [file ptr-28-54-s01.pdf]

Appendix 1

Standard Rehabilitation Program

ROM exercises

The following exercises were performed to maintain and improve the ROM, promote smooth joint movement, and prevent blood clots:

- Knee flexion-extension exercises (active and passive)
- Patellar mobilization (active and passive)
- Ankle dorsiflexion and plantarflexion exercises (active and passive)
- Incision site mobilization (active and passive)
- Massage and stretching of the peri-knee muscles (passive)
- Knee joint mobilization (as needed) (passive)

Muscle strengthening exercises

Muscle strengthening exercises included both weight-bearing and non-weight-bearing exercises. The program began with 1 set of 10 repetitions and gradually progressed to 3 sets of 10 repetitions. Set numbers and resistance were increased incrementally when the patient could complete 10 repetitions without pain or swelling. The specific exercises were as follows:

- Resistance exercises: quadriceps setting, seated knee extension, straight leg raise, side-lying hip abduction, and hip raise
- Body weight exercises: slow squats and step-ups

Walking exercise

Training focused on progressing from safe assisted walking with a cane to independent walking while relearning proper gait patterns:

- Instructions on gait improvement, including heel strike, toe-off, stride length, and proper use of walking aids
- Gradual progression from indoor to outdoor walking
- Outdoor walking was initiated at 5-min intervals, progressing up to 30 min, and included walking on flat surfaces, slopes, stairs, and uneven terrain

Activities of daily living exercise

Guidance and practice to safely perform activities of daily living when the patient has not yet acquired sufficient ROM and muscle strength.

Electrophysical agents

Icing or heat therapy was applied as needed to reduce pain and inflammation, with the leg elevated during the sessions.

Home Exercise Program

Up to 1 month after surgery

- ROM exercises: active-assisted movements, 5 repetitions every 2 h
- Strengthening exercises: quadriceps setting, seated knee extension, straight leg raises, side-lying hip abduction, and hip raises (sets were adjusted based on progress during outpatient physical therapy)
- Swelling management: elevate the legs as needed (approximately 20 min every 4 h) and apply ice

1 month after surgery

- Additional strengthening exercises: slow squats, sit-to-stand exercises, and step-up training
- Walking exercise: start with continuous 5-min walking sessions, gradually increasing the walking time and distance
