# Supplementary material for: Effects of Neuromuscular Electrical Stimulation on Quadriceps Muscle Strength in the Early Postoperative Period after Total Knee Arthroplasty
Source: Phys Ther Res. 2025 Mar 10;28(1):54–60. doi: 10.1298/ptr.E10327 (PMC12047048; doi:10.1298/ptr.E10327)
Supplement: Appendix 2. — Detailed results of analysis of variance for split-plot factorial design. [file ptr-28-54-s02.pdf]

Appendix 2

Detailed results of analysis of variance for split-plot factorial design

Pre: Preoperative Po: postoperative

- **Quadriceps strength**

Main effect Factor: p = 0.412 (η^2 0.010), Time: p = 0.000 (η^2 0.129)*

Interaction effect: p = 0.443 (η^2 0.003)

Multiple comparison for Time

Po4wks - Po8wks p = 0.000 Po4wks < Po8wks *

Po4wks - Po12wks p = 0.000 Po4wks < Po12wks *

Po8wks - Po12wks p = 0.000 Po8wks < Po12wks *

- **Knee flexion ROM**

Main effect Factor: p = 0.668 (η^2 0.002), Time: p = 0.000 (η^2 0.176)*

Interaction effect: p = 0.004 (η^2 0.024) *

Multiple comparison for factor

at Pre p = 0.084

at Po4wks p = 0.351

at Po8wks p = 0.297

at Po12wks p = 0.236

Multiple comparison for time

at NMES G.

Pre - Po4wks p = 0.000 Pre > Po4wks *

Pre - Po8wks p = 0.000 Pre > Po8wks *

Pre - Po12wks p = 0.000 Pre > Po12wks *

Po4wks - Po8wks p = 0.005 Po4wks < Po8wks *

Po4wks - Po12wks p = 0.000 Po4wks < Po12wks *

Po8wks - Po12wks p = 0.001 Po8wks < Po12wks *

at Con G.

Pre - Po4wks p = 0.000 Pre > Po4wks *

Pre - Po8wks p = 0.002 Pre > Po8wks *

Pre - Po12wks p = 0.232 Pre = Po12wks

Po4wks - Po8wks p = 0.001 Po4wks < Po8wks *

Po4wks - Po12wks p = 0.000 Po4wks < Po12wks *

Po8wks - Po12wks p = 0.002 Po8wks < Po12wks *

- **Knee extension ROM**

Main effect Factor: p = 1.000 (η^2 0.000), Time: p = 0.000 (η^2 0.069)*

Interaction effect: p = 0.292 (η^2 0.008)

Multiple comparison for Time

Pre - Po4wks p = 0.074 Pre = Po4wks

Pre - Po8wks p = 0.018 Pre < Po8wks *

Pre - Po12wks p = 0.000 Pre < Po12wks *

Po4wks - Po8wks p = 0.042 Po4wks < Po8wks *

Po4wks - Po12wks p = 0.001 Po4wks < Po12wks *

Po8wks - Po12wks p = 0.018 Po8wks < Po12wks *

- **VAS score**

Main effect Factor: p = 0.192 (η^2 0.025), Time: p = 0.000 (η^2 0.362)*

Interaction effect: p = 0.139 (η^2 0.028)

Multiple comparison for Time

Pre - Po4wks p = 0.000 Pre > Po4wks *

Pre - Po8wks p = 0.000 Pre > Po8wks *

Pre - Po12wks p = 0.000 Pre > Po12wks *

Po4wks - Po8wks p = 0.002 Po4wks > Po8wks *

Po4wks - Po12wks p = 0.000 Po4wks > Po12wks *

Po8wks - Po12wks p = 0.003 Po8wks > Po12wks *

- **TUG score**

Main effect Factor: p = 0.296 (η^2 0.018), Time: p = 0.000 (η^2 0.184)*

Interaction effect: p = 0.462 (η^2 0.009)

Multiple comparison for Time

Pre - Po4wks p = 0.001 Pre < Po4wks *

Pre - Po8wks p = 0.397 Pre = Po8wks

Pre - Po12wks p = 0.438 Pre = Po12wks

Po4wks - Po8wks p = 0.000 Po4wks > Po8wks *

Po4wks - Po12wks p = 0.000 Po4wks > Po12wks *

Po8wks - Po12wks p = 0.011 Po8wks > Po12wks *
